# Supplementary material for: Dengue Seroprevalence and Factors Associated with Dengue Seropositivity in Petaling District, Malaysia
Source: Int J Environ Res Public Health. 2022 Jun 11;19(12):7170. doi: 10.3390/ijerph19127170 (PMC9223214; doi:10.3390/ijerph19127170)
Supplement: Supplementary file 1 [file ijerph-19-07170-s001.zip › ijerph-1655111-supplementary S1.pdf]

Individual Questionnaire for Dengue Seroprevalence Study in Malaysia
Borang Soal-selidik Individu untuk Kajian Seroprevalens Denggi di Malaysia

Individual Research Identification Number: Date of interview:
Nombor Pengenalan Kajian Individu: *Tarikh Temubual:*

Age: year month Sex: Male / Female
Umur: ______ *tahun* ______ *bulan* *Jantina: Lelaki / Perempuan*

Ethnicity: Malay / Chinese / Indian / Others, specify
*Bangsa: Melayu / Cina / India / Lain-lain, nyatakan*___________

Marital Status: Single / Married / Divorced / Widowed
Status Perkahwinan: Bujang / Berkahwin / Bercerai / Janda & Duda

Completed Education: Primary/ Secondary / Tertiary / Informal education / No
Tamat Pendidikan: Rendah / Menengah / Pengajian Tinggi / Tidak Formal / Tiada

Occupation:
Pekerjaan: __________________

1. Have you ever had dengue fever before this?
Pernahkah anda menghidapi demam denggi sebelum ini?

Yes/*Ya* → to question 1a (*ke soalan 1a*)
No/*Tidak*)
Don't know/*Tidak tahu* } to question 2 (*ke soalan 2*)
Refuse/*Enggan jawab*)

1a. How many times did you have dengue fever before?
Berapa kalikah anda menghidapi demam denggi sebelum ini?

Where was the diagnosis (number -) made?
Dimanakah diagnosa demam denggi (kali ke-) diberi?

Have you ever been admitted to the ward for dengue fever (number -)? Where?
Pernahkah anda masuk ke wad untuk episod demam denggi (kali ke-)? Di mana?

Number *Bilangan*	When? *Bila?* (Months/Years) ago *(bulan/tahun) yang lalu*	Location of dengue diagnosis *Tempat diagnosa denggi dibuat*		Admission to ward *Kemasukan Wad*	
First *Pertama*		Clinik/Hospital *Klinik/Hospital*	Public/Private *Kerajaan/Swasta*	Yes/No *Ya/Tidak*	Public/Private *Kerajaan/Swasta*
Second *Kedua*		Clinik/Hospital *Klinik/Hospital*	Public/Private *Kerajaan/Swasta*	Yes/No *Ya/Tidak*	Public/Private *Kerajaan/Swasta*
Third *Ketiga*		Clinik/Hospital *Klinik/Hospital*	Public/Private *Kerajaan/Swasta*	Yes/No *Ya/Tidak*	Public/Private *Kerajaan/Swasta*
Fourth *Keempat*		Clinik/Hospital *Klinik/Hospital*	Public/Private *Kerajaan/Swasta*	Yes/No *Ya/Tidak*	Public/Private *Kerajaan/Swasta*

2. Have you ever had FEVER AND any of the symptoms below in the past 90 days? (Can tick more than one answer)
Pernahkah anda mengalami DEMAM DAN salah satu simptom di bawah dalam 90 hari yang lepas? (Boleh pilih lebih dari satu jawapan)

retroorbital eye pain<br>*sakit belakang mata*	
headache<br>*sakit kepala*	
generalised bodyache<br>*sakit badan merata*	
arthralgia/joints ache<br>*sakit sendi*	
myalgia/muscular ache<br>*sakit otot*	

Rash<br>*Ruam*	
Nausea<br>*Loya*	
Vomiting<br>*Muntah*	
Anorexia/Loss of appetite<br>*Kurang selera makan*	

If yes, when?/*Jika ya, bila?* ___________ days ago/ *hari yang lalu* → to Q2a (*ke S2a*)
No/*Tidak*)
Don't know/*Tidak tahu* } THE END/*TAMAT*
Refuse/*Enggan jawab*)

2a. Have you seen a doctor for this fever episode?
Pernahkah anda berjumpa dengan doctor untuk episod demam tersebut?

Yes/*Ya* → to Q2b (*ke S2b*)
No/*Tidak*)
Don't know/*Tidak tahu* } THE END/*TAMAT*
Refuse/*Enggan jawab*)

2b. Where did you get the doctor's advice?
Dimanakah anda mendapat nasihat doktor tersebut?

Public clinic/*Klinik kerajaan*)
Private clinic/*Klinik swasta*)
Public hospital/*Hospital kerajaan* } to Q2c (*ke S2c*)
Private hospital/*Hospital swasta*)
Others, specify/*Lain-lain, nyatakan*___________)
Don't know/*Tidak tahu*)
Refuse/*Enggan jawab* } THE END/*TAMAT*

2c. Was it diagnosed as dengue?
Adakah diagnosa denggi diberi?

Yes/*Ya* → to Q2d (*ke S2d*)
No/*Tidak*)
Don't know/*Tidak tahu* } THE END/*TAMAT*
Refuse/*Enggan jawab*)

2d. Were you admitted to the ward?
Adakah anda dimasukkan ke wad?

Yes/*Ya* → to Q2e (*ke S2e*)
No/*Tidak*)
Don't know/*Tidak tahu* } THE END/*TAMAT*
Refuse/*Enggan jawab*)

2e. Where were you admitted for this episode of dengue fever?
Di wad hospital manakah anda dirawati untuk episod demam denggi tersebut?

Public hospital/*Hospital kerajaan*)
Private hospital/*Hospital swasta*)
Don't know/*Tidak tahu* } THE END/*TAMAT*
Refuse/*Enggan jawab*)

3a. If you have fever, when do you seek treatment from clinic/hospital? / *Kebiasaanya, pada hari ke berapakah anda ke klinik/hospital apabila demam?*

1. First day of fever / *Hari pertama demam*
2. Second day of fever / *Hari kedua demam*
3. Third day of fever / *Hari ketiga demam*
4. Fourth day of fever / *Hari keempat demam*
5. More than five days of fever / *Lebih daripada lima hari*
6. Do not go to clinic or hospital / *Tidak pergi ke klinik atau hospital*

3b. If you do not seek treatment from clinic/hospital, What do you usually do? / *Jika anda tidak pergi ke klinik atau hospital apakah yang biasanya anda lakukan?*

1. No action taken / *Tidak buat sebarang tindakan*
2. Take panadol / *Makan panadol*
3. Seek alternative treatment / *Dapatkan rawatan alternatif*

Commercial RDT Serial Number:
Nombor Siri "RDT Komersial"

Test<br>*Ujian*	Positive<br>*Positif*	Negative<br>*Negatif*	Equivocal<br>*Samar*
NS1			
IgG			
IgM			

VIRO-Track Catridge Serial Number:
Nombor Siri Katrij VIRO-Track:

Test<br>*Ujian*	Positive<br>*Positif*	Negative<br>*Negatif*	Value<br>*Nilai*
IgG			
IgM			

4.0 Dengue Risk Factor

Please tick which ever applicable / *Sila tandakan mana-mana yang berkenaan*:

#	Item / *Perkara*	Yes / *Ya*	No / *Tidak*	Not applicable / *Tidak berkenaan*
4.1.	Screened windows / *Tingkap yang berpelindung*			
4.2.	Screened doors / *Pintu berpelindung*			
4.3.	Types of mosquito control measure / *Penggunaan jenis kawalan nyamuk*			
4.4a.	Bed net / *Kelambu*			
4.4b.	*Mosquito coil, mat, liquid vapouriser* / Lingkaran nyamuk, tikar atau cecair penghalau nyamuk			
4.4c.	Insecticide aerosol spray */ Semburan aerosol racun serangga*			
4.5.	Use air conditioner at home / *Menggunakan penyaman udara di rumah*			
4.6.	Use mosquito repellent cream or spray / *Menggunakan repelen atau krim penghalau nyamuk*			

4.7. How many persons live in the household / *Berapa orang tinggal di dalam rumah ini*?

1- 1
2- 2-4
3- 5-9
4- >=10

4.8. Number of indoor potted plants / *Jumlah tumbuhan berpasu di dalam rumah?*
1- Tiada/ *None*
2- >=1

5. When is the active biting time for Aedes mosquitoes? / *Pada waktu bilakah nyamuk Aedes aktif menggigit?*

Read out the following options, the respondent only needs to choose one out of them / *Bacakan pilihan jawapan, responden hanya perlu pilih satu sahaja*

1. Early morning (6-8am) / *Awal pagi (6-8am)*
2. Noon / *Tengah hari*
3. Evening/dusk (6-8pm) / *Petang/senja (6-8pm*)
4. Early morning (6-8am) & evening/dusk (6-8pm) / *Awal pagi (6-8am) & Petang/senja/ (6-8pm)*
5. Midnight / *Tengah malam*
-7 Do not know / *Tidak tahu*
-9 Refuse to answer / *Enggan jawab*

6. For the past 12 months is there any fogging activity in your neighbourhood carried out by the Health Department/local Authority? / *Dalam tempoh 12 bulan yang lepas, adakah kakitangan Pejabat Kesihatan/Pihak Berkuasa tempatan melaksanakan semburan kabus di kejiranan anda?*

1. Yes / *Ya*
2. No / *Tidak*
-7 Do not know / *Tidak tahu*
-9 Refuse to answer / *Enggan jawab*

7. For the past 12 months, is there any activity on search and destroy of Aedes mosquito breeding sites carried out in your neighborhood by the community? / *Dalam tempoh 12 bulan yang lepas, adakah kejiranan anda melaksanakan aktiviti mencari dan memusnah tempat pembiakkan nyamuk Aedes secara bergotong royong?*

1. Yes / *Ya*
2. No / *Tidak*
-7 Do not know / *Tidak tahu*
-9 Refuse to answer / *Enggan jawab*

7.1 If yes, did you participate? / *Jika ya, adakah anda sertai?*

1. Yes / *Ya*
2. No / *Tidak*
-7 Do not know / *Tidak tahu*
-9 Refuse to answer / *Enggan jawab*

8. Do you use larvicide? / *Adakah anda pernah menggunakan racun pembunuh jentik-jentik?*

1. Yes / *Ya*
2. No / *Tidak*
-7 Do not know / *Tidak tahu*
-9 Refuse to answer / *Enggan jawab*

8.1 Where do you usually get your larvicide? */ Kebiasaannya, dimanakah anda mendapatkan bekalan racun pembunuh jentik-jentik tersebut?*

1. Given by health department/Local Authority staff / *Diberi oleh kakitangan pejabat kesihatan/pihak berkuasa tempatan*
2. Get it for free from government health facilities / *Diperolehi secara percuma dari fasiliti kesihatan kerajaan*
3. Buy from the supermarket / *Beli dari Pasaraya*
4. Buy from the hardware shop / *Beli dari kedai perkakasan rumah*
5. Buy from petrol station / *Beli dari stesyen minyak*
6. Others, please specify / *Lain-lain, nyatakan*

-7 Do not know / *Tidak tahu*
-9 Enggan jawab / *Refuse to answer*

8.2 Where do you apply the larvicide? */ Di manakah anda bubuh/masukkan racun pembunuh jentik-jentik tersebut?*

(More than one answer is accepted) / ***(Lebih dari satu jawapan diterima)***

1. Water storage container / *Bekas simpanan air*
2. Vase/container for aquatic plants / *Pasu tumbuhan akuatik*
3. Flower pot liner / *Alas pasu bunga*
4. Vase/container for fresh flowers / *Bekas bunga segar*
5. Others, Please specify / *Lain-lain, sila nyatakan* ………………..

-7 Do not know / *Tidak tahu*
-9 Refuse to answer / *Enggan jawab*

9. Do you check and eliminate stagnant water in the house properly? / *Adakah anda ada memeriksa dan membuang air bertakung di dalam rumah?*

1- Yes, during epidemics / *Ya, semasa berlaku wabak*
2- Yes, everyday / *Ya, setiap hari*
3- Yes, at least once a week / *Ya, sekurang-kurangnya sekali seminggu*
4- No */ Tidak*

9.1 Do you check and eliminate stagnant water outside and surrounding of your home properly? / *Adakah anda ada memeriksa dan membuang air bertakung di sekeliling luar rumah?*

1- Yes, during epidemics / *Ya, semasa berlaku wabak*
2- Yes, everyday / *Ya, setiap hari*
3- Yes, at least once a week / *Ya, sekurang-kurangnya sekali seminggu*
4- No */ Tidak*
